# Prevalence and Influencing Factors of Mixed Dentition Malocclusion in Children Aged 6–12 Years in Jinzhou, China

**DOI:** 10.3290/j.ohpd.b4100913

**Published:** 2023-05-17

**Authors:** Jianhui Xu, Xin Li, Xiaoyu Liu, Siwei Li, Yibing Lu

**Affiliations:** a Dentist, Second Affiliated Hospital of Jinzhou Medical University, Jinzhou, Liaoning Province, China. Idea, wrote the manuscript, performed the experiments, performed statistical evaluation, experimental design.; b Professor, Second Affiliated Hospital of Jinzhou Medical University, Jinzhou, Liaoning Province, China. Proofread the manuscript, contributed substantially to discussion.; c Dentist, Second Affiliated Hospital of Jinzhou Medical University, Jinzhou, Liaoning Province, China. Performed the experiments in partial, experimental design.; d Dentist, Second Affiliated Hospital of Jinzhou Medical University, Jinzhou, Liaoning Province, China. Performed the experiments in partial, experimental design.; e Lecturer, Second Affiliated Hospital of Jinzhou Medical University, Jinzhou, Liaoning Province, China. Performed the experiments, experimental design.

**Keywords:** children, epidemiological investigation, malocclusion, risk factors

## Abstract

**Purpose:**

To investigate the prevalence, clinical manifestations and related risk factors of malocclusion in schoolchildren of Jinzhou City, China.

**Materials and Methods:**

A total of 2162 children aged 6–12 years were randomly selected from various districts of Jinzhou. Conventional clinical examination was performed by stomatologists, and the results were described based on different clinical manifestations of malocclusion and individual normal occlusion. Further, a questionnaire survey completed by children’s parents or guardians provided the demographic data, lifestyle, and oral habits. The distribution of individual normal occlusion and malocclusion was documented in percentage, and Pearson’s Χ^2^ was used for two-factor analysis. The data were statistically analysed using SPSS software (version 25.0) with a significance level of α = 0.05.

**Results:**

A total of 1129 boys and 1033 girls were included in this study, i.e. 52.2% and 47.8% of the total number of children, respectively. The prevalence of malocclusion in children aged 6–12 years old in Jinzhou was 67.9%, of which crowded dentition was the most common form, with a prevalence of 71.8%, followed by deep overbite, anterior crossbite, dental spacing, deep overjet, anterior edge-to-edge occlusion, and anterior open bite. In the logistic regression model, the results showed that BMI index had little effect on the occurrence of malocclusion (p > 0.05), while dental caries, bad oral habits, retained primary teeth, and a low labial frenum were all related to the occurrence of malocclusion (p < 0.05). Moreover, the higher frequency and duration of bad oral habits were associated with a higher likelihood of malocclusion.

**Conclusions:**

The prevalence of malocclusion in children aged 6–12 years in Jinzhou is high. In addition, bad oral habits (such as lip biting, tongue thrusting, biting/gnawing objects, unilateral chin supporting, and unilateral mastication) and other related risk factors (such as dental caries, mouth breathing, retention of primary teeth, and low labial frenum, etc) were associated with malocclusion.

Malocclusion refers to tooth, jaw, and craniofacial deformities and is mainly manifested as individual tooth dislocation, abnormal arch shape and tooth arrangement, and abnormal relationship of the dental arches, jaw, and craniofacial structure. General statistical results have shown that about 60.0% to 70.0% of malocclusion cases are related to acquired factors, mainly including bad oral habits, abnormal oral function, and irregularities or disturbances in the process of dental replacement.^9^

Malocclusion can not only cause abnormal position of teeth and jaws but also movement disturbance of mandibular opening and closing, for instance, severe anterior occlusive occlusion, obvious occlusal interference, and occlusal trauma. Malocclusion can affect the appearance of the face, e.g. through an irregular arrangement of teeth or abnormal arrangement of dental arch and jaw, and sometimes cause psychological abnormalities.^12^ Malocclusion is harmful to the oral occlusal system in many ways and sometimes has a severe impact on development and function. Therefore, the goal of oral correction of malocclusion is to restore the balance, function, and appearance of the whole occlusal system. The standard of orthodontic treatment is individual normal occlusion, not ideal normal occlusion. That is, any slight malocclusion that does not interfere with the physiological function can be included in the category of normal occlusion. Because individual occlusion within the normal category differs from person to person, it is called individual normal occlusion. The ideal normal occlusion was put forward by Angle, meaning that the whole pair of teeth is preserved, the teeth are arranged neatly on the maxillary and mandibular dental arches, the apical fossa relationship between the maxillary and mandibular teeth is completely correct, and the occlusal relationship between the maxillary and mandibular dental arches is ideal. After treatment of malocclusion, the craniofacial morphology, function, and profile of teeth and jaws have achieved a new balance and coordination.^27^ The results of the national survey published by Fu Minkui in 2000 show that malocclusion in Chinese children is as high as 71.2% in the mixed dentition stage and 72.9% in the initial stage of permanent teeth.^6^

The prevalence of malocclusion increases significantly from primary dentition to mixed dentition. Bad oral habits are one of the main causes of malocclusion.^7^ However, the malocclusion of permanent dentition is closely related to the existence and development of early primary-dentition and mixed-dentition malocclusion. Malocclusion can affect the development of occlusal, maxillofacial, and oral health function, as well as appearance. It can be systemically detrimental and even cause serious psychological disorders.^13^

The purpose of this study is to investigate the prevalence of malocclusion in the selected population, including prevalence, clinical manifestations, and related risk factors, and to provide a theoretical foundation for the formulation of prevention and treatment strategies.

## Materials and Methods

### Ethics Approval and Consent to Participate

The study protocol was designed in compliance with the Helsinki Declaration and approved by the Ethics Committee of the Second Affiliated Hospital of Jinzhou Medical University. The parents/guardians of participants signed the informed consent.

### Study Design and Sample

This was a cross-sectional study in oral preventive medicine. From September to November 2021, a multi-stage, cluster, and random sampling method was used to investigate the prevalence of malocclusion among children aged 6 to 12 years in Guta District, Taihe District, Linghe District, Linghai City, Beizhen City, Yi County and Heishan County of Jinzhou City through school examination. First, one district, one city, and one county were selected in Jinzhou City using simple random sampling. In this manner, Guta District, Linghai City, and Yi County were chosen. After that, in the above three sample districts and counties, each district and county randomly selected three streets or townships, for a total of nine streets (townships). Finally, using simple random sampling, one school was selected from each street or township, and a total of nine schools were obtained: Jiefang Primary School, Baodi Primary School, Beihu Primary School, Jiqing Primary School, Fulun Primary School, Songshan Primary School, Huaxing Primary School, Labor Insurance Primary School, and Zhenjiangtai Primary School. In each school, cluster sampling was used to investigate children aged 6 to 12 years, with equal numbers of boys and girls.

In the sample size estimation formula N = K×Q/P, K is determined by the allowable error of the research project. Here, the allowable error is taken as 10.0%, so that K = 400. According to the national malocclusion epidemiological survey organised by the CSA (Chinese Stomatological Association) in 2000, the prevalence of the mixed dentition stage was 71.2%.^6^ Taking the participants lost to follow-up into account, individual samples did not meet the inclusion criteria, the sample size was increased by 10.0%, and a total of 2200 people were finally selected.

To be included, the following criteria had to be met: 1. children’s age between 6 and 12 years; 2. place of residence Jinzhou City; 3. consent to actively participate and cooperate with the questionnaire survey and clinical examination was given; 4. informed consent to participate in the survey was given; 5. children were healthy and free of mental or any other diseases. Further, the following were excluded from the study: 1. children who were not in school on the day of the examination; 2. children who were still uncooperative with the examination after behavioural management; 3. children who were receiving or had received orthodontic treatment; 4. children with local or systemic problems, or trauma affecting facial development; 5. children with cleft lip and palate; and 6. children without erupted first permanent molars.

### Clinical Examination and Questionnaire

Clinical examination by stomatologists was performed under natural light. The stomatologists used conventional oral examination instruments (cotton swabs, flashlights, etc.) to record the examination results based on different clinical manifestations of malocclusion and individual normal occlusion (Table 1). With reference to the Fourth National Oral Health Questionnaire (students), our study developed the Oral Health Questionnaire of the Second Affiliated Hospital of Jinzhou Medical University to record general information about the subjects, including sex, age, ethnic groups, parents’ educational background, etc. The questionnaire also contained items on bad oral habits, including types (lip biting, tongue thrusting, biting/gnawing objects [e.g. fingernails, pencils, clothing, etc], unilateral chin supporting, sucking fingers, and unilateral mastication), their duration and frequency, as well as related risk factors, such as caries, mouth breathing, BMI index, retention of primary teeth and low labial frenum.

**Table 1:** Types of malocclusion and related diagnostic criteria

Types of malocclusion		Diagnostic criteria
Deep overjet	I	The maximum horizontal distance from the cutting edge of the maxillary incisor to the labial surface of the mandibular anterior tooth is < 5 mm.
	II	The maximum horizontal distance from the cutting edge of the maxillary incisor to the labial surface of the mandibular anterior tooth is 5 to 8 mm.
		The maximum horizontal distance from the maxillary incisor to the labial surface of the mandibular anterior teeth is > 8 mm
Deep overbite	I	The labial face of the crown of the mandibular anterior teeth is covered by the crown of the maxillary anterior teeth, or the incisal edge of the mandibular anterior teeth bite on the lingual incisor of the maxillary anterior teeth more than 1/3 to 1/2.
	II	The crown of the maxillary anterior teeth covers 1/2 to 2/3 of the labial surface of the mandibular anterior teeth, or the cutting edge of the mandibular anterior teeth engages between 1/2 and 2/3 of the cutting edge of the lingual surface of the maxillary anterior teeth or at the lingual carina.
	III	The crown of the maxillary anterior teeth covers more than 2/3 of the labial surface of the mandibular anterior teeth, and even bites down on the labial gingival tissue of the mandibular anterior teeth, or the cutting edge of the mandibular anterior teeth bites down on the lingual gingival tissue or hard palate mucosa of the maxillary anterior teeth.
Anterior open bite	I	The vertical separation of maxillary and mandibular incisors is within 3 mm.
	II	The maxillary and mandibular incisors are vertically separated by 3 to 5 mm.
	III	The maxillary and mandibular incisors are vertically separated by more than 5 mm.
Anterior crossbite		In the case of apical malocclusion, the mandibular anterior teeth bite down on the labial side of the maxillary anterior teeth, covering a negative value.
Anterior edge-to-edge occlusion		In the case of apical malocclusion, the incisal margin of the maxillary and mandibular teeth contact, overlie and cover the anterior teeth with zero occlusal relationship.
Partial occlusion		The occlusal plane is skewed and the dentition or jaw is asymmetrical.
Crowding		The difference between the sum of the crown width and the length of the existing arc of the dental arch, the degree of crowding ≥ 2 mm.
Spacing		The tooth size is relatively less than the bone mass, which is characterised by gaps between teeth.

### Occlusion Criteria Judgement

The diagnostic criteria for each type of malocclusion are described based on different clinical manifestations of malocclusion and individual normal occlusion,^27^ as shown in Table 1. Normal occlusion can be further described as the median occlusal position, where the mesial buccal tip of the maxillary first permanent molar engages in the mesial buccal groove of the mandibular first permanent molar, but with a slight malocclusion deformity in the dental arch, which did not greatly hinder the physiological process. This kind of individual occlusion within the normal category is termed ‘individual normal occlusion’.^7,27^

### Statistical Analysis

The data were entered into Microsoft Excel by double input, and the database was established. The statistical analysis of the data was carried out by SPSS software, version 25.0 (IBM; Armonk, NY, USA) with a significance level of α = 0.05. The Χ^2^ test was used for bivariate analysis, and statistically significant variables were included in the binary logistic regression analysis. The odds ratio (OR) and 95% confidence interval (CI) were calculated to distinguish risk factors associated with malocclusion.

### Reliability Test

The survey included an on-site principal, three clinical examiners, three recorders, two questionnaire investigators, and two data-entry personnel. In order to avoid examiner bias, the three examiners repeated the examination during the inspection. To test reliability in the early stage of this epidemiological investigation, all participants received uniform clinical training on malocclusion. Before the final examination, the reliability of the examiner was evaluated by conducting reliability tests on 20 subjects (including one reference examiner and three candidates). Kappa coefficients within the 3 inspectors were 0.90, 0.92, and 0.87, respectively, while kappa values between inspectors were 0.80. During the investigation, 5.0% of the subjects were selected for the second reliability test, and the kappa coefficient was greater than 0.85.

## Results

The datasets used and/or analysed during the current study are available from the corresponding author upon reasonable request.

### Differences of Malocclusion by Sex, Age, Ethnic Groups, Urban and Rural Areas

Among the 2200 participants, 15 pupils did not complete the questionnaire survey, seven pupils’ parents did not sign the informed consent forms, and 16 pupils were excluded for not meeting the inclusion criteria. Finally, 2162 participants received a complete clinical examination and a questionnaire, with a participation rate of 98.3%.

Among those 2162 children, 1129 were males, accounting for 52.2%, of which 768 cases suffered from malocclusion with a prevalence of 68.0%. 1033 were females, accounting for 47.8% of the total, including 700 children with malocclusion, with a prevalence of 67.8%. There was no statistically significant difference between the sexes (p > 0.05). However, there were statistically significant differences in the prevalence of malocclusion among the seven age groups from 6 to 12 years old (p < 0.05). The prevalence of malocclusion increased with age in children 6 to 8 years old, from 69.9% (6 years), 75.1%(7 years), to 73.8% (8 years old). In contrast, malocclusion was seen in 65.1% of 9-year-old children, 64.7% of 10-year-old children, 64.3% of 11-year-old children, and 65.7% of 12-year-old children.

The survey found no differences in the prevalence of malocclusion among children from different ethnic groups, regions, and economic backgrounds in Jinzhou (p > 0.05) (Table 2).

**Table 2:** Comparison of the prevalence of malocclusion by sex, age, ethnic group and area of residence

		Total (n = 2162)	Malocclusion (n = 1468)	Normal (n = 694)	X^2^	p
		n (%)	n (%)	n (%)		
Gender						
	Male	1129 (52.2)	768 (68.0)	361 (32.0)	0.017	0.897
	Female	1033 (47.8)	700 (67.8)	333 (32.2)		
Age						
	6	102 (4.7)	71 (4.8)	31 (4.5)	18.726	0.005
	7	289 (13.4)	217 (14.8)	72 (10.4)		
	8	351 (16.2)	259 (17.6)	92 (13.3)		
	9	447 (20.7)	291 (19.8)	156 (22.5)		
	10	430 (19.9)	278 (18.9)	152 (21.9)		
	11	339 (15.6)	218 (14.9)	121 (17.4)		
	12	204 (9.4)	134 (9.1)	70 (10.1)		
Nationality						
	Han	1752 (81.0)	1182 (80.5)	570 (82.1)	1.057	0.590
	Man	355 (16.4)	246 (16.8)	109 (15.7)		
	Other	55 (2.5)	40 (2.7)	15 (2.2)		
Area						
	Urban	1925 (89.0)	1308 (89.1)	617 (88.9)	0.019	0.892
	Rural	237 (11.0)	160 (10.9)	77 (11.1)		

### Distribution of Common Clinical Manifestations of Malocclusion

**Fig 1:**
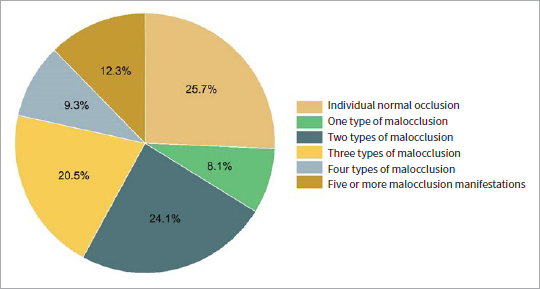
The severity of malocclusion in the study population.

Table 3 shows the prevalence of typical clinical manifestations of malocclusion, with crowded dentition being the most prevalent (71.8%), followed by deep overbite, anterior crossbite, dental spacing, deep overjet, edge-to-edge occlusion, and anterior open bite. The prevalences were 39.3%, 16.1%, 12.1%, 8.9%, 4.0%, and 2.1%, respectively (Table 3). Except for 694 (32.1%) individual normal occlusions, 8.1% of the children had only one type of malocclusion. Most children (47.5%) showed two to four types of malocclusion simultaneously, while 12.3% had five or more malocclusion types. The severity of malocclusion in the subjects is shown in Fig 1.

**Table 3:** Distribution of common clinical manifestations of malocclusion

Clinical symptoms		Total n (%)		Male n (%)		Female n (%)		X^2^	p
Crowded dentition									
	Normal	610	28.2	297	26.3	313	30.3	4.248	0.039
	Crowded	1552	71.8	832	73.7	720	69.7		
Deep overbite									
	Normal	1312	60.7	661	58.6	651	63.0	4.524	0.033
	Deep overbite	850	39.3	468	41.5	382	37.0		
Deep overjet									
	Normal	1752	81.0	899	79.6	853	82.6	3.049	0.081
	Deep overjet	410	19.0	230	20.4	180	17.4		
Dental spacing									
	Normal	1901	87.9	991	45.8	910	88.1	0.051	0.822
	Dental spacing	261	12.1	138	6.4	123	11.9		
Crossbite									
	Normal	1814	83.9	942	83.4	872	84.4	0.382	0.537
	Crossbite	348	16.1	187	16.6	161	15.6		
Anterior open bite									
	Normal	2117	97.9	1104	97.8	1013	98.1	0.205	0.651
	Anterior open bite	45	2.1	25	2.2	20	1.9		
Anterior edge-to-edge occlusion									
	Normal	2075	96.0	1085	96.1	990	95.8	0.098	0.754
	Anterior edge-to-edge occlusion	87	4.0	44	3.9	43	4.2		

**Fig 2:**
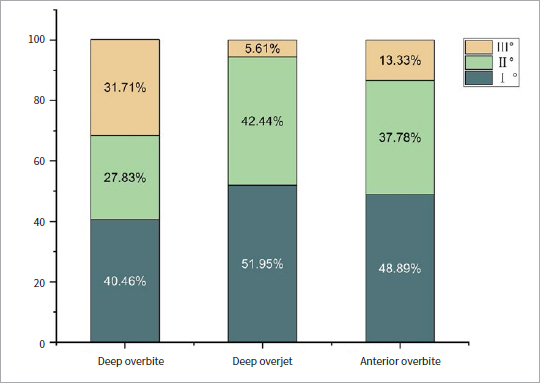
Distribution of different degrees of deep overbite, deep overjet and anterior open bite.

The distribution of various degrees of deep overbite, deep overjet, and anterior open bite is shown in Fig 2, among which 1st-degree deep overbite, 1st-degree deep overjet, and 1st-degree anterior open bite accounted for the largest proportion in their respective classification, with proportions of 40.5%, 52.1%, and 48.9%, respectively.

### Analysis of Risk Factors Related to Malocclusion

**Fig 3:**
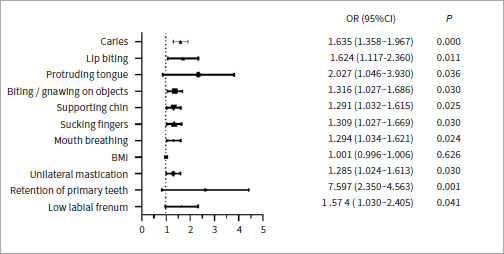
Visual analysis of risk factors of malocclusion by binary logistic regression.

In terms of the association between bad oral habits (lip biting, tongue thrusting, biting/gnawing on objects, unilateral chin supporting, sucking fingers, and unilateral mastication), related risk factors (caries, mouth breathing, BMI index, retention of primary teeth and low labial frenum) and malocclusion, no statistically significant difference between BMI and the incidence of malocclusion (p > 0.05) was found. However, statistically significant differences did exist between malocclusion and bad oral habits and other related risk factors (p < 0.05) (Fig 3).

Regarding the frequency and duration of the bad oral habits mentioned above, it was found that the higher the frequency and duration of bad oral habits, the greater was the possibility of malocclusion (Table 4). The differences were statistically significant (p < 0.05).

**Table 4:** Relationship between the duration and frequency of bad oral habits and malocclusion

		Malocclusion (n)	Individual normal occlusion (n)	Total (n)	Ratio (%)	X^2^	p
Duration							
	Never	1165	586	1751	66.5	10.172	0.006
	More than one month	69	26	95	72.6		
	More than one year	234	82	316	74.1		
Frequency							
	Never	1143	569	1712	66.8	8.553	0.036
	Lasting a few minutes a day	228	99	327	69.7		
	Lasting tens of minutes a day	46	14	60	76.7		
	Lasting several hours a day	51	12	63	81.0		

## Discussion

The purpose of this study was to provide detailed insight into the prevalence and related risk factors of malocclusion in children aged 6 to 12 in Jinzhou City. An effort was made to ensure the accuracy of oral habit diagnostics, based not only on a questionnaire for parents but also on clinical observation. For results showing consistency, the STROBE Statement checklist was used.^22^

In recent years, many studies have been published on the prevalence of malocclusion, some of which were related to the prevalence of malocclusion in children with mixed dentition. Perhaps due to the differences in race, geographical environment, eating habits, and economic status, the prevalences of malocclusion in the same age group differ according to region and country, e.g. 46.5% in Japan,^15^ 83.3% in India,^3^ and 87.0% in Iran.^29^ In China, Fu Minkui et al found that malocclusion of children with mixed dentition was as high as 71.2%.^6^ In the past decade, survey results have varied greatly across the country, for instance, with 79.4% in Shanghai^26^ and 69.4% in Hailing District, Taizhou City.^11^

The results of this survey showed that the prevalence of malocclusion in children aged 6 to 12 years old in Jinzhou was 67.9%, which corresponds well with international and national reports.^14,23,25^ The pathology and related factors of malocclusion are complex. It is known that genetic factors are highly correlated with craniofacial abnormalities.^19^ In addition to genetic factors, environmental factors can also affect the normal development of occlusion. For example, finger sucking can cause anterior teeth to develop an open bite.^8^

According to the existing literature, bad oral habits are closely related to the occurrence of malocclusion, and the prevalence of malocclusion in children with bad oral habits is higher than that in children without bad oral habits. Bad habits such as lip biting, finger sucking, and tongue thrusting will lead to malocclusion such as tilted teeth, narrow dental arch, anterior open bite, etc.^17^ The results of this survey substantiate this. The prevalence of malocclusion in children with bad oral habits (sucking fingers, mouth breathing, tongue thrusting, etc) is higher than that in children without bad oral habits, and the difference is statistically significant (p > 0.05).

The current study found no statistically significant difference in the prevalence of malocclusion between boys and girls, consistent with results found by other authors.^27^ Previous data indicate differences in the prevalence of malocclusion among different age groups, with the prevalence increases with age. The prevalence in primary dentition is the lowest, followed by the period of mixed dentition, with the highest occurring in the early stage of permanent dentition.^1,21^ As this study included subjects with mixed dentition, the prevalence of malocclusion was higher in children aged 6 to 8 years and lower in children aged 9 to 12 years. This could be due to the fact that the children from 6 to 8 years old were in the early stage of mixed dentition. With increasing age, the jaws continue to develop, their length and width increasing. It is beneficial to adjust the position of permanent teeth and improve the symptoms of malocclusion.^5^ In addition, the results of this investigation demonstrated that the prevalence of malocclusion in children with mixed dentition tends to stabilise after the age of 9 years. This is mainly associated with the eruption of the first permanent molar, central incisor, and lateral incisor. Children with erupted central and lateral incisors had a lower prevalence and type of malocclusion than children in whom these teeth had not yet erupted, which is consistent with the findings of Diao et al.^4^ However, further longitudinal studies are needed to confirm these findings. There was no difference in prevalence between urban and rural areas, which is consistent with the results of a recent survey.^4^ Moreover, there was no difference in the prevalence between different ethnic groups, which differs from some other studies conducted in China.^4,26^

The survey found that the prevalence of crowding was the highest among local abnormalities (71.2%), and was related to unbalanced degenerative changes in the craniofacial region.^16^ The phylogenetically determined degenerative imbalance between the jaw and teeth results in the tooth mass being greater than bone mass (crowding). The result of this survey shows that the prevalence of spacing is 12.1%. The gaps in the primary and mixed dentition facilitate eruption and the establishment of the permanent dentition. Nevertheless, it was found that 9 of the 16 subjects did not have primary-tooth spacing. In spite of this, their permanent dentition is open, indicating that it may not be necessary to use developmental and primary space to solve the problem of crowding in the permanent dentition. The relationship between crowding, spacing, and malocclusion needs to be confirmed by other longitudinal data.^2,4,18^

Compared with the national average, Jinzhou children not only have a high prevalence of malocclusion but also have complex and diverse types of malocclusion.^10,24,26^ The survey found that except for 694 (32.1%) individual normal occlusions, 8.1% of the children had only one type of malocclusion. Most children (47.5%) showed two to four kinds of malocclusion simultaneously, and 12.3% of the children had five or more malocclusion types simultaneously. Complex and diverse manifestations of malocclusion increase the treatment difficulty and treatment time of malocclusion; thus, early prevention and treatment of malocclusion is important in children. At the same time, Jinzhou City should strengthen its official support for and financial investment in preventing and treating children’s malocclusion.

It is unclear whether there is a correlation between caries and malocclusion. Some scholars^28^ believe that caries is a risk factor for malocclusion, as caries can change the height and width of the crown, promote crowding and changes in occlusal surfaces, and affect the temporomandibular joints. The prevalence of malocclusion in children with carious primary teeth is 2.04 times higher than in children with caries-free primary teeth.^13,28^ However, some authors have pointed out that the occurrence of malocclusion is closely related to the time and severity of caries, and that only caries in mixed and permanent dentition may lead to malocclusion.^20^

This cross-sectional study on the relationship between caries and malocclusion in children aged 6-12 years in Jinzhou found a statistically significant correlation between the two (p < 0.05). Future epidemiological studies on malocclusion need a standardised examination protocol, a larger and more systematic sample size, and control of other variables to analyse the relationship between a dependent variable and malocclusion.

This study has some limitations, as follows:

1. The cross-sectional study design cannot explain the causal relationship between local oral disorders and malocclusion; we can only make assumptions about the aetiology of malocclusion.
2. The sample size of 6-year-old children selected in this study is small, and the representativeness of the sample is limited, which may limit the transferability of some of the conclusions of this paper.
3. The children who were still uncooperative with the examination after behavioural management (which is intended to enable children to cooperate with oral examination through comfort, encouragement, and communication) may not be able to carry out daily oral health care, possibly exacerbating malocclusion due to an unfavourable intraoral environment Such individuals were excluded from the survey, which might have distorted the results.
4. For the investigation of local oral and developmental disorders, only the occurrence of oral disorders was recorded, restricting the content of the investigation. At the same time, the relationship between local oral and developmental disorders and a specific type of malocclusion could not be explored, which may affect the validity of the data.
5. Although there was strict quality control, measurement bias among the examiners was inevitable and had a certain impact on the results.

There are few reports on malocclusion in the mixed dentition period in China. The number of subjects investigated in previous studies is low, so the results may be susceptible to large errors. In addition, due to the small sample size or survey index, most of these studies do not represent the prevalence in specific areas. Through the oral health examination, the prevalence and related risk factors of malocclusion in children aged 6-12 years in Jinzhou were studied, and the risk factors related to malocclusion were further confirmed. At the same time, the investigation process is helpful to strengthen policies promoting early oral health education and spread the knowledge of prevention and treatment of malocclusion among the general population to reduce the prevalence of malocclusion.

## Conclusion

The prevalence of malocclusion in children aged 6-12 years in Jinzhou remains higher than in other regions of China. Additionally, the prevalence of malocclusion is closely to related to risk factors such as bad oral habits (tongue thrusting, lip biting, etc), caries, and a low labial frenum. Therefore, it is paramount to monitor the development of malocclusion during early stages of development and perform any necessary interventions on its influencing factors to reduce the prevalence of malocclusion and other adverse reactions.
